# Leishmaniac Quest for Developing a Novel Vaccine Platform. Is a Roadmap for Its Advances Provided by the Mad Dash to Produce Vaccines for COVID-19?

**DOI:** 10.3390/vaccines10020248

**Published:** 2022-02-07

**Authors:** Kwang Poo Chang, Joseph M. Reynolds, Ying Liu, Johnny J. He

**Affiliations:** Department of Microbiology/Immunology, Center for Cancer Cell Biology, Immunology and Infection, Chicago Medical School, Rosalind Franklin University of Medicine and Science, North Chicago, IL 60064, USA; joseph.reynolds@rosalindfranklin.edu (J.M.R.); ying.liu@rosalindfranklin.edu (Y.L.); johnny.he@rosalindfranklin.edu (J.J.H.)

**Keywords:** *Leishmania*, Leishmaniasis, SARS-CoV-2, COVID-19, Singlet oxygen, cell inactivation, whole-cell vaccine platform

## Abstract

“Bugs as drugs” in medicine encompasses the use of microbes to enhance the efficacy of vaccination, such as the delivery of vaccines by *Leishmania*—the protozoan etiological agent of leishmaniasis. This novel approach is appraised in light of the successful development of vaccines for Covid-19. All relevant aspects of this pandemic are summarized to provide the necessary framework in contrast to leishmaniasis. The presentation is in a side-by-side matching format with particular emphasis on vaccines. The comparative approach makes it possible to highlight the timeframe of the vaccine workflows condensed by the caveats of pandemic urgency and, at the same time, provides the background of *Leishmania* behind its use as a vaccine carrier. Previous studies in support of the latter are summarized as follows. Leishmaniasis confers life-long immunity on patients after cure, suggesting the effective vaccination is achievable with whole-cell *Leishmania*. A new strategy was developed to inactivate these cells in vitro, rendering them non-viable, hence non-disease causing, albeit retaining their immunogenicity and adjuvanticity. This was achieved by installing a dual suicidal mechanism in *Leishmania* for singlet oxygen (^1^O_2_)-initiated inactivation. In vitro cultured *Leishmania* were genetically engineered for cytosolic accumulation of UV-sensitive uroporphyrin I and further loaded endosomally with a red light-sensitive cationic phthalocyanine. Exposing these doubly dye-loaded *Leishmania* to light triggers intracellular production of highly reactive but extremely short-lived ^1^O_2_, resulting in their rapid and complete inactivation. Immunization of susceptible animals with such inactivated *Leishmania* elicited immunity to protect them against experimental leishmaniasis. Significantly, the inactivated *Leishmania* was shown to effectively deliver transgenically add-on ovalbumin (OVA) to antigen-presenting cells (APC), wherein OVA epitopes were processed appropriately for presentation with MHC molecules to activate epitope-specific CD8+ T cells. Application of this approach to deliver cancer vaccine candidates, e.g., enolase-1, was shown to suppress tumor development in mouse models. A similar approach is predicted to elicit lasting immunity against infectious diseases, including complementation of the spike protein-based vaccines in use for COVID-19. This pandemic is devastating, but brings to light the necessity of considering many facets of the disease in developing vaccination programs. Closer collaboration is essential among those in diverse disciplinary areas to provide the roadmap toward greater success in the future. Highlighted herein are several specific issues of vaccinology and new approaches worthy of consideration due to the pandemic.


**Table of Content**

Page1. Introduction32. Information/database search3–43. Results (SARS-CoV-2/COVID-19 versus *Leishmania*/leishmaniasis)
 3.1. Causative agents

  3.1.1. Molecular genetics4

3.1.2. Primary and secondary infection of host cell5–6

3.1.3. Species and variants6–7
3.2. Signs, symptoms and sequelae of the disease


3.2.1. The major clinical symptoms7

3.2.2. Associated clinical consequences7–8
3.3. Epidemiology 


3.3.1. Transmission8

3.3.2. Distribution8–9

3.3.3. Incidence9

3.3.4. Risk factors9
3.4. Surveillance, treatment and control


3.4.1. Diagnosis9–10

3.4.2. Clinical managements



A. Chemotherapy10


B. Alternative therapy 10


C. Immunotherapy10–11

3.4.3. Preventive measures11

3.4.4. Control programs11–12
3.5. Vaccine


3.5.1. Development, production and application



A1-A6 vaccine platforms for COVID-1912–14


B1-B4 vaccine platforms for human leishmaniasis13–15

3.5.2. Clinical trials15

3.5.3. Human challenge trials15–16

3.5.4. Vaccine rollout16

3.5.5. Socio-economic impacts on vaccination rate & herd immunity16

3.5.6. Human immune response to vaccination17

3.5.7. Rare, but serious side-effect of live adenovirus vaccines?17
3.6. Leishmania platform for safe and effective delivery of vaccines


3.6.1. *Leishmania* as an effective vaccine platform17–18

3.6.2. *Leishmania* as a safe vaccine platform by installation of a dual


  suicidal mechanism for singlet oxygen-initiated inactivation18–20

3.6.3. Leishmania delivery of vaccines for T-cell immunity against



infectious & malignant diseases20–224. Discussion

4.1. Prospect of disease control from the perspective of COVID-19 pandemic 22–23
4.2. The success of anti-Covid vaccines stimulates new approaches 23
4.3. *Leishmania* vs adenovirus as a vaccine platform23
4.4. *Leishmania* delivery of vaccines against multiple diseases24
4.5. *Leishmania* platform for anti-Covid vaccines24
4.6. Assessing human immune response to pathogens/vaccines25–265. Conclusion266. References27–30

## 1. Introduction

Vaccinology, or the science of vaccines, has received a great deal of attention since the beginning of 2020. This is due completely to the prominent role it has played in response to the emergence of COVID-19—the most severe pandemic since the Spanish flu of 1918. There have been tremendous advances with unprecedented rapidity in all fields of vaccinology, including the basic science of etiological agents, vaccine designs, delivery strategies, and biotechnologies, scale-up manufacturing, clinical trials, rollout strategies, evaluation of human immune response, epidemiology database and analysis, and public health and policies.

It is an opportune time to assess the question of what we have learned from this unprecedented and overwhelming effort for future implementation in the interest of controlling infectious diseases by vaccination. This is particularly relevant to diseases, which are not as sweeping as COVID-19 but are still significant and widespread to cause localized epidemics. One example among many is leishmaniasis. This disease has been reported to occur for centuries until today, inflicting considerable morbidity and mortality on the human population worldwide, especially in resource-poor countries. One distinctive feature of leishmaniasis is the development of lasting immunity for patients after cure. The causative agents are thus useful, when rendered safe for use, not only as a whole-cell vaccine against this disease but also as a carrier with adjuvant activities or adjuvanticity for effective delivery of foreign protein vaccines against other diseases including COVID-19.

In this context, we begin by comparing COVID-19 and leishmaniasis in areas with common threads of relevance to vaccinology, i.e., etiological agents, infection and immunity, clinical diseases and managements, epidemiology, control programs, public health, and other areas. Special emphasis is placed on all aspects pertaining to vaccines. The contrast between the two by this comparative approach delineates the success of COVID-19 vaccine development and also provides the rationale of using *Leishmania* as a vaccine delivery vehicle. The prospects of advancing *Leishmania*-based vaccinology along with others are discussed from the perspectives of this comparative analysis.

## 2. Information/Database Search

Information and the citation of literature for *Leishmania* and leishmaniasis are based on our familiarity with these subjects, as summarized in recent reviews [1,2]. For SARS-CoV-2 and COVID-19, PubMed was searched, yielding a very large body of literature, from which relevant articles were selectively cited. Specific facts and figures mentioned came from several informative sources, including, but not limited to the authoritative websites listed below (and the search tools included therein). Each website cited was followed by the date of its first posting without further update or that of the last update at the time of its access during the preparation of this review.

https://www.who.int/publications/m/item/draft-landscape-of-covid-19-candidate-vaccines, 21 January 2022;

https://www.who.int/neglected_diseases/Ending-the-neglect-to-attain-the-SDGs--NTD-Roadmap.pdf, 29 January 2021;

https://www.niaid.nih.gov, 8 September 2020;

https://www.cdc.gov/coronavirus/2019-ncov/vaccines/keythingstoknow.html?s_cid=10490:covid%h, 12 January 2022;

https://covid19.nih.gov, 20 January 2022;

https://coronavirus.jhu.edu, 22 November 2021;

https://www.nytimes.com/interactive/2020/science/coronavirus-vaccine-tracker.html?smid=rd, 18 January 2022;

https://www.nytimes.com/interactive/2020/science/coronavirus-drugs-treatments.html?smid=rd, 12 January 2022;

https://www.fda.gov/emergency-preparedness-and-response/coronavirus-disease-2019-covid-19/covid-19-vaccines, 13 January 2022;

https://biology.mit.edu/video-post/britt-glaunsinger-coronavirus-biology/https://www.youtube.com/watch?v=8_bOhZd6ieM, 8 September 2020.

## 3. Results (SARS-CoV-2/COVID-19 vs. *Leishmania*/Leishmaniasis)

### 3.1. Causative Agents

#### 3.1.1. Molecular Genetics

SARS-CoV-2 is an RNA virus of ~100 nm in diameter and is the causative agent of the COVID-19 pandemic. The genome is a negative strand RNA of ~30 kb, giving rise to a positive copy of the template for RNA-dependent RNA polymerase (RDRP)-mediated transcription after cell entry [3]. Two large transcripts are generated from the 5′ part of the viral genome using the host transcription/translation machinery to produce polypeptides, 1a and 1ab. The latter contains a bit of the former, resulting from a frame-shift translation mechanism on ribosomes. Both are cleaved proteolytically into functional proteins needed for viral replicative/transcriptional functions. The 3′ part of the viral genome is subjected to discontinuous transcription, which stops at 6 different locations, yielding transcripts of different lengths that merge with the leader sequence from the very 5′ end. The transcripts from these subgenomic RNAs produce largely accessory or structural proteins needed to assemble mature virions for extracellular exit. A total of ~27 proteins are produced by the unique mechanisms described from 14 open reading frames (ORF) of the viral RNA genome.

*Leishmania* are single-cell eukaryotic protozoa, each 2 to 5 um in diameter in the intracellular stage, causing endemic and sometimes epidemic leishmaniasis. The haploid genome size of these largely diploid cells is in the order of up to 55 mb. *Leishmania* genomic replication, transcription, and translations share unique features with other trypanosomatid protozoa (see literature cited in [1,2]). Each cell contains up to 36 pairs of chromosomes, ranging in size from 30 to >3500 kb. Each chromosome is subjected to Pol II-mediated transcription polycistronically, starting from one or more transcription-initiation or inversion sites, which do not function as promoters. The polycistronic transcripts produced are trans-spliced into functional mRNAs with 5′- and 3′-UTRs via the activities of spliceosomes resting on the intergenic regions. Transcription is regulated by the acquisition of miniexon of 107 nt to cap the 5′ end of the spliced mRNAs, and also by the length of their 3′ UTR after splicing for stability. Production of transcripts for abundant proteins relies on gene dosage effect, i.e., the presence of many head-to-tail tandemly repeated gene clusters, e.g., miniexon genes. Such gene clusters are in the chromosomes of both *Leishmania* spp. and related *Trypanosoma* spp. in synteny, indicative of their evolution from a common ancestral origin for functional significance. The open reading frames (ORF) of their genes contain no intron for intron/exon cis splicing. There are up to 8000 ORFs in the *Leishmania* genome, encoding discrete proteins per cell. Many have no sequence-homologs in other eukaryotes and thus are functionally undefined. *Leishmania* and other trypanosomatid protozoa each have a single but highly branched mitochondrion. Each mitochondrial genome consists of ~20 copies of maxicircles (10–20 kb each) and 10^4^–10^5^ copies of minicircles (~1 kb each), which are concatenated and localized in a specialized region of the mitochondrion known as kinetoplast. Transcripts of cryptic genes from the maxicircles undergo extensive RNA editing to create functional mRNAs. Guide RNAs from both circles provide the templates for precise editing. This is a subject of enormous interest but will not be further discussed herein.

Both SARS-CoV-2 and *Leishmania* are obligate intracellular pathogens of mammalian cells. Convergent evolution appears to account for the common features they share for the presence of intron-less ORFs and discontinuous transcription. There are very large and obvious differences between the two in the genome size, complexity of genome replication, transcription, and translation, reflective of relative autonomy for *Leishmania* and completely host-dependence of the coronavirus.

#### 3.1.2. Primary and Secondary Infection of Host Cells

SARS-CoV-2: The life cycle of this virus in their host cells has been under intensive investigation [3,4] (see the video by Britt Glaunsinger). Briefly, the virion’s spike proteins latch to the angiotensin-converting enzyme 2 (ACE-2) receptors for cell entry by endocytosis. All cells, which bear ACE-2 receptors, are thus theoretically susceptible to infection by this and other related coronaviruses. Viral entry is followed by an uncoating of the virions that involves proteolytic cleavage of participating proteins and membrane fusion events leading to the release of viral genomic payloads into the cytoplasm for activities as described in Section 3.1.1. Several viral proteins from cleavages of the polypeptides are needed to create double membrane vacuoles from rough ER for housing viral replicative and transcriptional complex (RTC). Other viral proteins, including RNA-dependent RNA polymerase, utilize host cell machinery to produce the positive genomic transcripts for translation. Additional viral proteins are produced, e.g., NSP (non-structural protein)-1 to shut down host cell mRNA translations, including interferon (INF), to inhibit its signaling activities. The structural proteins produced from the discontinuous transcription of the subgenomic RNAs are assembled on the RER membrane. Viral membrane, envelope, and nucleocapsid proteins are aligned for packaging the minus strand of the RNA genome produced. Mature virions are then put together for budding off from the membrane into the RER lumen and pass through Golgi for exit from cells by exocytosis via the egress pathway. The details of this infective cycle have been examined in the host cells of coronavirus-targeted organs, e.g., pneumocytes, alveolar macrophages, and nasal mucous cells [5]. The timeframe for each viral life cycle is estimated to include ~10 min for cell entry and ~10 h to complete the described intracellular events, yielding 10^3^ virions from each infected cell (more than one million virions per person per day). The virions released from the host cells infect additional susceptible cells, accounting for the spread of their infection. Death of SARS-CoV-2-infected cells via apoptotic and/or necrotic pathway is predicted from previous work on related coronavirus [6]. There has been no clear evidence for the presence of lysogenic stage for coronaviruses, as reported for Herpes viruses.

*Leishmania* take residence exclusively in mononuclear phagocytes or macrophages, as noted by examining infected tissues from leishmaniasis patients. There is a large body of literature on the study of how *Leishmania* infect macrophages in vitro [1,2]. Briefly, macrophages take up *Leishmania* also by receptor-mediated endocytosis. The C3bi-receptor is thought to be the major one, but almost all known receptors of macrophages have been reported to play some roles, depending on *Leishmania*-macrophage combinations used for the studies. *Leishmania* ligands for receptor-binding are their surface glycoconjugates [7], including a Zn-metalloprotease of 63 kd (gp63) and a unique lipophosphoglycans (LPG). The roles of additional surface and secretory molecules have been reported to facilitate the infection. The fusion of *Leishmania*-containing phagosomes with secondary lysosomes was observed, providing the initial evidence that *Leishmania* are phagolysosomal parasites of macrophages—immune cells whose very function is to pick up invading pathogens for elimination by phagocytosis. This is unexpected but has been substantiated by investigations during the subsequent decades for all *Leishmania* species examined in in vitro and in vivo models. It is also consistent with the ability of some species to grow in vitro continuously, in the absence of macrophages, under acidic conditions of pH 5 at the body temperatures of their mammalian hosts. One recent study found *Leishmania* localized in the early endosomes by using Rab markers to trace the dynamic of vesicular transport and membrane fusion [8]. There is no question that *Leishmania* live in a modified endosome-lysosome vacuolar system or parasitophorous vacuoles of macrophages. As such, *Leishmania* are warded off from the host cell cytoplasm by a membrane barrier, limiting host-parasite molecular exchanges. On the other hand, fluorescence-tagged macromolecules in the extracellular milieu of infected macrophages were found to enter the parasitophorous vacuoles via the endocytic pathway. Clearly, while *Leishmania* are intracellular, they remain accessible to soluble and particulate matters present in the extracellular environment and endocytosed by the host cells. Indeed, evidence indicates that infected macrophages ingest hemoglobin, providing heme as an essential nutrient to *Leishmania*, which are aerobic cells, but defective in biosynthesis for heme needed for respiration. Clearly, *Leishmania* do not depend on the host cell machinery for replication, transcription, and translation. The spread of *Leishmania* from one host cell to another is not as well-defined as the viral life cycle of infection. There are two possible pathways to account for the spread of *Leishmania* infection in vivo. Heavily infected macrophages replete with *Leishmania* may simply bust open, releasing them to infect additional macrophages. The infected macrophages may become degenerated in other incidences, but not lysed. They are thus recognized by macrophages freshly arrived at the scene as senescent cells for disposal along with *Leishmania* therein via scavenging phagocytosis. This mechanism of secondary infection is advantageous to *Leishmania* by sparing them from the danger of exposure to the lytic factors present in the host body fluids. *Leishmania* replicate asynchronously at a slow and variable rate in macrophages, making it difficult to estimate the time-lines for the progression of its intracellular life cycle.

Both SARS-CoV-2 and *Leishmania* establish infection in their respective host cells via receptor-mediated endocytosis. The mechanism for receptor-ligand specificity is well-defined for the coronavirus, accounting for its entry into a variety of ACE-2 receptor-bearing cells. In contrast, the resident host cells of *Leishmania* are specific and limited to macrophages (also dendritic cells for brief residence), but the mechanisms are haphazard, involving multiple ligand-receptor interactions. The coronavirus ends up in the cytoplasm, taking over the host biosynthetic machinery for reproduction. In contrast, *Leishmania* live independently in the vacuolar system of degradative pathways of their host cells. These differences account for their varying levels of molecular integration with their respective host cells and in the strategies of their extracellular exit for spreading.

#### 3.1.3. Species and Variants

SARS-CoV-2 belongs to the family Coronaviridae, which is subdivided into two subfamilies: Coronavirinae and Torovirinae [9]. There are many species of both, created by mutations during their genomic RNA replications in the host cells. They are mostly non-pathogenic or cause very mild enteric or respiratory diseases in humans. Exceptions are those causing more severe diseases such as the COVID-19 pandemic (under discussion), Middle East Respiratory Syndrome (MERS), and Severe Acute Respiratory Syndrome (SARS). More contagious and/or more virulent mutants of SARS-CoV-2 have emerged during the COVID-19 pandemic, e.g., Alpha variant B.1.1.7 (UK first report), Beta variant B.1.351 (S. Africa), Gamma variant P.1 (Brazil), Ipsilon variants B.1.427/B.1.429 (California), Iota variant B.1.526 (New York), B.1.617 double mutant of S. Africa + California, Delta variant B.1.617.2 (India), and Omicron variant (Botswana/Southern Africa). The first three have fewer than two dozen mutations in each, many of which are in the spike protein, responsible for the cell entry of the virus. Some of these mutants are associated with an increased infection of the upper respiratory tract, e.g., Eek (E484K). Other variants appear to interfere with effective immune response, such as the Beta variant. The Omicron variant that emerged at the time of this writing (November 2021) was designated by WHO as a variant of concern with >50 mutations, of which >30 are in the spike protein and others in immunologically important parts of the sequences. These mutations appear to enhance the viral transmissibility or contagiousness and to reduce the efficacy of the vaccines in current use. A variant reported earlier in Angola has many mutations, but they do not seem to increase the disease severity, i.e., hospitalization, fatality, and reinfection rates. US-CDC provides up-to-date information as mutants emerge worldwide (https://www.cdc.gov/coronavirus/2019-ncov/variants/, 11 August 2021). Note: The date here and below after each website cited is the date found on the webpage at the time of its first posting without further update or its last update when accessed by the authors during the preparation of this review).

*Leishmania* is a genus in the family of Trypanosoamtidae, consisting of two subgenera: *Leishmania* and *Viannia*. Some 20 species have been described to cause human cutaneous, mucocutaneous and visceral leishmaniasis. Member species of subgenus *Leishmania* are present worldwide (e.g., *L. major*, *L. tropica*, *L. donovani*, *L. infantum*, *L. mexicana*), while those of the *Viannia* group are limited to Central and South America (e.g., *L. braziliensis*, *L. panamensis*, *L. guianensis).*

SARS-CoV-2 is prone to mutations during replications as an RNA virus. Many mutations are neutral, some are lethal, and others are associated with an increase in viral transmission and disease severity. *Leishmania* genomes are much larger and relatively stable as diploid eukaryotes. In the absence of typical meiosis, genetic recombination is characterized as an infrequent occurrence based on an undefined mechanism. Overall, the phylogenetic evolution of *Leishmania* spp. is not well-understood in relation to their pathological phenotypes, although the diseases are well-defined as clinical entities in most geographical locations.

### 3.2. Signs, Symptoms, and Sequelae of the Disease

#### 3.2.1. The Major Clinical Symptoms

COVID-19 is largely an acute infectious disease. Clinical signs and symptoms emerge 2-14 days after infection, including fever or chills, cough, shortness of breath/difficulty breathing, fatigue, muscle or body aches, headache, loss of taste or smell, sore throat, congestion, runny nose, nausea or vomiting, and diarrhea. Young patients may be asymptomatic or experience a very mild form of the disease, but this may no longer be true for recently evolved new variants. Some of the symptoms listed may persist in some patients for months with additional neurological, cardiovascular, gastrointestinal medical problems known as “long haulers” [10]. Fatal cases are due primarily to respiratory paralysis as a result of pulmonary functional failure, mainly among older patients or those with underlying health problems, e.g., diabetes, cancer, and immunocompromised individuals. Multi-organ inflammatory syndromes (MIS-C) have been reported in some infected children [11,12,13].

Leishmaniasis is a chronic parasitic disease. The onset of clinical manifestations is slow and becomes noticeable weeks or months after infection. The disease is marked by a gradual progression to chronicity lasting for months or years. Asymptomatic cases are common. The severity of the disease depends on the sites of infection or location of infected macrophages, i.e., their confinement to the skin or systemic spreading to internal organs of RES, i.e., spleen, liver, and bone marrow. Simple cutaneous leishmaniasis is marked by the appearance of skin lesions as small papule or plaque, which may remain small, closed, and dry or evolve into wet and open volcanic lesions. Development of secondary or satellite lesions and recrudescence of apparently healed lesions are common, but only in some specific geographic locations. All cutaneous lesions are eventually self-healed spontaneously, indicative of effective immune mechanisms for clearance in most endemic areas. Mucocutaneous leishmaniasis also begins with skin infection, but it metastasizes into oropharyngeal and nasal cavities, causing non-healing lesions, with tissue damages and cartilage erosions, resulting in facial disfigurements. All types of tegumentary leishmaniases are non-fatal, but can cause considerable discomforts and morbidity, especially in mucocutaneous cases. Visceral leishmaniasis is an often fatal disease if not treated. Major clinical signs and symptoms arise from inflammatory disorders of hematopoietic and hematological systems, such as fever, lethargy, fatigue, weight loss, cachexia, anemia, lymphadenopathy, leukocytopenia, and hyperimmunoglobulinemia leading to hepatosplenomegaly and bone marrow dysfunctions.

#### 3.2.2. Associated Clinical Consequences

Patients of both diseases suffer from mild to severe mental disorders with clinical consequences. The practices of mask wearing, social distancing, lockdown, and isolation by quarantine are known to cause depression and behavioral disorders [14]. More serious is the post-Covid psychosis reported in a few cases. Patients of cutaneous leishmaniasis have been reported to suffer mentally due to prolonged suffering from a non-healing state and facial disfiguration [15].

Both pathogens cause diseases with a spectrum of clinical manifestations, ranging from asymptomatic to severe illness to death. Fatality is due to acute ARDS in COVID-19 and chronic RES disorders in visceral leishmaniasis. All forms of cutaneous leishmaniasis are non-fatal, and simple cutaneous leishmaniasis is most innocuous. Contributions to the clinical variations are due in part to different variants of the virus and different species of *Leishmania*.

### 3.3. Epidemiology

#### 3.3.1. Transmission

COVID-19 is an air-borne, highly transmissible infectious disease. Human-to-human transmission of SARS-CoV-2 occurs through direct, indirect, or close contact with infected individuals via their saliva or respiratory fomites, aerosol, droplets expelled by coughs, sneezes, talking, or singing. The virus appears to have jumped from bat to human via an intermediate animal of unknown species [16,17]. This view is weakened by the discovery of a bat coronavirus, which infects human cells efficiently by possessing a spike protein with receptor-binding domain (RBD) essentially identical to that of SARS-CoV-2 [18]. It is thus uncertain if COVID-19 is a zoonosis with a reservoir animal to keep the virus in circulation [19]. SARS-CoV-2 has been reported to infect many domesticated and captive animals, from pets (cats and dogs) to zoo animals, e.g., pumas, tigers, gorillas, and snow leopards, to farmed mink. Outbreaks in mink farms have already shown that the virus underwent mutations and that the infected animals passed the variants back to humans [20]. The risk of animals spreading COVID-19 to people is nevertheless still considered to be low (https://www.cdc.gov/coronavirus/2019-ncov/daily-life-coping/animals.html, 18 November 2021). Infection of the deer populations with human SARS-CoV-2 at high rates raised the alarm in many States, e.g., ~80% of the white-tailed deer population infected in Iowa (https://www.the-scientist.com/news-opinion/researchers-detect-coronavirus-in-iowa-deer-69376, 3 November 2021). Worrisome are the outbreaks of COVID-19 in the unregulated mink farms of the USA with the potential spreading of the disease to the wild animals (https://www.nytimes.com/2022/01/19/magazine/spillback-animal-disease.html, 19 January 2022).

Leishmaniasis has long been known largely as a vector-borne zoonotic disease. The blood-feeding females of phlebotomine sand flies transmit leishmaniasis from animals to animals, from animals to humans, from humans to humans, and possibly from humans to animals. The diseases are largely a zoonosis of canines, rodents, and edentates. Humans are incidental dead-end hosts, except in some places where anthroponosis is indicated by the apparent absence of animal reservoirs. Hares and raccoon dogs have been found to harbor pathogenic visceral *Leishmania* and are thus potential reservoirs. In humans, transmissions via the congenital route, blood transfusion, coitus, organ transplantation, and other means of contact have been reported, but their contribution to epidemiology is probably small. Needle-sharing among intravenous drug users contributes to person-to-person transmission.

Zoonotic leishmaniasis with wild animals as the reservoir is inherently difficult to eradicate. Prevention is the best control method via prophylactic vaccination of the at-risk human population.

#### 3.3.2. Distribution

COVID-19 has been reported in >220 countries and territories (https://www.worldometers.info/coronavirus/countries-where-coronavirus-has-spread/, 25 January 2022). In contrast to other infectious diseases, COVID-19 hits affluent countries as hard as or even harder than the resource-poor countries of the world.

Leishmaniasis is a very widespread disease, existing in all continents except Antarctica. In Australia, leishmanial infection has been reported to occur only in kangaroo. Mediterranean basin is a well-known endemic area where the visceral disease is now rare in Southern Europe, except for canine leishmaniasis. World Health Organization provided the following information (https://www.who.int/news-room/fact-sheets/detail/leishmaniasis, 8 January 2022): In 2020, more than 90% of new cases for visceral leishmaniasis (VL) occurred in 10 countries: Brazil, China, Ethiopia, Eritrea, India, Kenya, Nepal, Somalia, South Sudan, Sudan and Yemen. About 95% of cutaneous leishmaniasis (CL) cases occur in the Americas, the Mediterranean Basin, the Middle East, and Central Asia. In 2020 > 85% of new CL cases occurred in 10 countries: Afghanistan, Algeria, Brazil, Colombia, Iraq, Libya, Pakistan, Peru, the Syrian Arab Republic, and Tunisia. The distribution generally coincides with that of the vector sand flies in resource-limited countries of temperate and tropical areas.

#### 3.3.3. Incidence

COVID-19: Its clinical incidences and consequences have been closely watched worldwide as a pandemic. According to the Center for Systems Science and Engineering (CSSE) at Johns Hopkins University, as of 27 January 2022, there are 364 million confirmed cases with >5.6 million death in >200 countries/territories and >73 million cases with >877,000 deaths in the USA.

Leishmaniasis: As a neglected disease, its incidences have not been closely tracked. Annually, the numbers of new cases worldwide are estimated to be 50,000 to 90,000 for VL and 600,000 to 1 million for CL, respectively, according to WHO (https://www.who.int/news-room/fact-sheets/detail/leishmaniasis, 8 January 2022). These are very rough estimates and are thought to be under-counted by 25–45% of the actual incidences. A world population of up to one billion is at risk of leishmaniasis in approximately 100 countries [1,2].

#### 3.3.4. Risk Factors

COVID-19**:** The risk increases with exposure to the virus-contaminated air and with age for severe illness, ranging from requiring hospitalization and intensive care to death, older adults being at the highest risk. People of any age are at risk of severe illness, especially those with certain underlying medical conditions, e.g., heart or lung problems, weakened immune systems, severe obesity, diabetes, and possibly pregnancy [21].

Leishmaniasis: The risk increases with human activities that increase the exposure of individuals to infected sand fly vectors in the endemic areas. Environmental and climate changes are associated with the risk of leishmaniasis. Epidemics thus often flare up sporadically in endemic regions where forest or arid lands are reclaimed for urbanization and agricultural or industrial uses in developing countries. Risk factors further include metabolic diseases, such as obesity and immunosuppressive conditions that are congenital or produced in cancer treatments and, as required, in organ transplantation or infection. HIV co-infection was a significant risk factor, in southern Europe initially, and is now a concern in other countries, such as Brazil and India. Additional risk factors include natural disasters, such as earthquakes, famine, drought, floods, and the man-made upheaval of war.

### 3.4. Surveillance, Treatment, and Control

#### 3.4.1. Diagnosis

COVID-19: All available strategies and technologies have been mobilized to rapidly develop diagnostics with variable specificity and sensitivity for COVID-19, including nasal swap and saliva samples for viral RNA detection by RT-PCR, and nasal/blood samples for detection of viral antigens or antibodies quantitatively by ELISA and qualitatively by dipsticks. Food and Drug Administration (FDA) has granted Emergency Use Authorization (EUA) for many diagnostics, including those for self-tests at home. Rollouts of diagnostic tests vary with the strategies of pandemic controls. Under the policy of “live with the pandemic” in the western world, diagnosis of the population has been based on making the kits and tests freely available. Under the “zero Covid policy” in China, massive populations have been mobilized in the millions for repeated daily tests.

Leishmaniasis: Diagnosis of leishmaniasis is limited to those in the endemic areas, relying largely on the microscopic visualization of parasites in infected tissues obtained from diseased skin or organs (bone marrow, spleen). Samples collections from the latter are painful and risky to the patients for acquiring secondary infection and injuries. The biopsied samples are directly examined microscopically or after amplification by cultivation for the presence of *Leishmania*. Noninvasive diagnosis means are experimentally available to detect either *Leishmania*-specific antigens or antibodies in saliva or urine. Less invasive diagnosis to detect specific DNA by PCR or antigens/antibodies by ELISA is not widely available, except for visceral leishmaniasis using the rK39 dipstick for the presence of antibodies specific to the *Leishmania*- kinesin 39 aa repeats.

#### 3.4.2. Clinical Managements

##### A. Chemotherapy

COVID-19: Chemotherapy is currently unsatisfactory. Remdesivir is the antiviral in use to help some patients with mild disease recover sooner. It does not work for all such patients and often does not benefit severely ill patients. Dexamethasone and similar steroids are the only medicines in use to increase the survival rate of patients sick enough to require extra oxygen in ICU. Molnupiravir is another antiviral in clinical trials, showing similar effectiveness or the lack of it. The outcome of its clinical trials released subsequently by Merck showed clear-cut benefits if taken early, prompting FDA to issue EUA for its clinical application. Both antivirals are nucleoside drugs to block viral replication [22]. Shionogi announced human trials of a 3CL protease inhibitor [23] as the first once-a-day pill for COVID-19 patients. Nirmatrelvir/Ritonavir tablets (PAXLOVID™) (Pfizer) with similar properties gained FDA’s EUA. The difficulty of developing antiviral compounds by blocking virus-specific polymerases and proteases has been discussed [24]. The antimalarial drug amodiaquine may be re-purposed for use against COVID-19. It was found highly effective at preventing viral entry, as determined by microfluidic Lung Airway Chip or human organ chip microfluidic culture technology [25].

Leishmaniasis: Current chemotherapy of this disease relies on the use of antiquated drugs, e.g., pentavalent antimonials (sodium stibogluconate, Glucantime, or meglumine antimoniate), amidine compounds (pentamidine), or amphotericin B, which are either not effective or toxic or difficult to administer. The last compound proves effective in liposome format for treating visceral leishmaniasis, but it is not cost-effective for patients in resource-poor countries. Additional anti-leishmanial drugs include miltefosine as an orally effective drug and aminoglycoside paromomycin for topical or internal applications. Chemotherapy of leishmaniasis has become increasingly difficult, resulting not only from using a handful of already unsatisfactory drugs but also because of the development of drug resistance. A combination of different drugs, each at a low dose, is the current approach attempting to circumvent this problem.

##### B. Alternative Therapy

COVID-19: Current clinical management of severe COVID-19 includes supportive care, such as supplemental oxygen and mechanical ventilatory support when indicated.

Cutaneous leishmaniasis: Many alternative therapies have been tried but not universally adopted for treating lesions, i.e., thermotherapy, cryotherapy, photodynamic therapy, and maggot therapy. The major obstacle to assessing their effectiveness is the self-healing tendency of these lesions.

##### C. Immunotherapy

COVID-19: Emergency Use Authorization by FDA for the treatment of COVID-19 includes medications with anti-spike protein monoclonal antibodies, e.g., Casirivimab with Imdevimab cocktails of Regeneron, and Bamlanivimab/Etesevimab of Eli Lilly, and Sotrovimab of GSK-Vir Biotech. Initial trials proved effective in vitro and safe in a small number of subjects. The EUAs for the first two were withdrawn for their ineffectiveness in treating patients infected with the Omicron variant. Monoclonal antibody drugs are generally expensive. COVID-19 convalescent sera/plasma therapy was highly promoted [26] and heavily supported by US government-sponsored programs, including plasma collections by Red Cross/Blood bank and extensive uses in many hospitals and medical centers. These programs were scaled back substantially and limited to clinical trials to define the effective range of antibody titers in the plasma, stages of COVID-19, and viral variants [27]. Extensive mutations of the Omicron variant in its spike proteins render it insusceptible to inhibition by antibodies in the plasma/serum from patients caused by earlier variants.

Leishmaniasis: Immunotherapy of leishmaniasis has been the subject of review articles, but both clinical and experimental trials are limited in scope.

#### 3.4.3. Preventive Measures

COVID-19: For the highly transmissible air-borne COVID-19, the strategies of barriers and isolation are preventive measures, including mask-wearing, hand sanitization, social distancing, travel restriction, and different scales of lockdown.

Leishmaniasis: As a vector-born endemic disease, preventive measures are based on different strategies to avoid sand fly bites, i.e., the use of pesticides, insect repellants for avoidance, and bed nets as a barrier. Dog collards with insect repellants are used in places where canines are the proven reservoir animals.

#### 3.4.4. Control Programs

COVID-19: A remarkable program was set up to fight COVID-19 with tremendous resources of unprecedented scale, i.e., Operation Warp Speed. It was a public/private partnership initiated by the US government to facilitate and accelerate the development, manufacturing, distribution of COVID-19 vaccines, therapeutics, and diagnostics (https://en.wikipedia.org/wiki/Operation_Warp_Speed, 13 January 2022). It was officially inaugurated on 15 May 2020 and funded with about $10 billion from the CARES Act (Coronavirus Aid, Relief, and Economic Security) passed by the US Congress on 27 March 2020. This is an interagency program involving US-CDC, -FDA, -NIH-NIAID, -BARDA of DHHS, -DOD, -USDA, DE (Department of Energy), and DVA to prioritize COVID-19 for investigation and coordination to expedite all medical, administrative, and logistic needs. Seven biotech/drug companies were chosen to receive funding of >1 billion each in most cases to develop and produce vaccines or therapeutics. The investments yielded two mRNA (Pfizer-BioNTech, Pleasant Prairie, WI, USA; Moderna, Cambridge, MA, USA) and one adenovirus-delivered (Johnson & Johnson, New Brunswick, NJ, USA) vaccine within 1 year time starting from laboratory design to shot-in-the arm inoculation. This is a feat accomplished with amazing rapidity, although the safety and effectiveness of all new vaccines cannot be determined until a large database is established from very wide use for many years. This is also true for all the other vaccines in the pipeline of this program. A spike peptide + adjuvant vaccine (Novavax, Gaithersburg, MD, USA) has cleared Phase III clinical trial. Another adeno-vaccine (AstraZeneca, London, UK/Oxford University, Oxford UK) was successfully produced and authorized for use in >120 countries. The remainders were either in trouble of significant delay (Sinofi, Paris, France/GSK, Brentford, UK) or abandoned altogether because of ineffectiveness (Merck, Durham, NC, USA/IAVI, New York, NY, USA). Progress has been made more slowly for the diagnostics and therapeutics supported by this program. FDA granted EUA (November 2021) for pills of molnupiravir by Merck and Paxlovid (a protease inhibitor) by Pfizer to be taken orally at the first signs of COVID-19 to stave off its severe clinical consequences based on unpublished data of clinical trials by the respective drug companies.

Leishmaniasis: National programs were previously launched to control visceral leishmaniasis. One started in 1950 in China, consisting of patient identification for treatments, control of vector sand flies, and elimination of dogs as the reservoir. The program was successfully executed by the allocation of sufficient resources to mobilize medical, public health specialists, and the general public for strict and persistent enforcement of its prescribed measures. Methodical surveillance during the subsequent years showed a total elimination of the disease in a large formally endemic area of eastern China. Today, visceral leishmaniasis occurs sporadically in the western region of Sichuan, Gansu, and Xinjiang. A similar kala-azar elimination program was launched in 2014 to control visceral leishmaniasis in the highly endemic area of India. The program has successfully reduced caseloads significantly. Neither program matches the resources and technologies of Operation Warp Speed for the pandemic COVID-19.

### 3.5. Vaccine

#### 3.5.1. Development, Production, and Application

A. COVID-19 vaccines: The extremely short timeframe for developing multiple formats was made possible chiefly by enormous government funding to provide tremendous incentives to the private sectors. Included in the support are not only vaccine designs and scale-up production before proof of safety and efficacy but also regulatory expedition of the reviews to grant emergency use authorization (EUA). Numerous articles have been published about the development and production of vaccines for COVID-19 [28]. As of 21st January 2022, WHO lists 334 vaccines in development, of which >190 are pre-clinical and >140 clinical. Listed below are examples of five different formats, of which the first four target spike protein (see Section 3.1.2) as the mono-specific vaccines and three of the five (1, 2, and 5) have already been used widely for vaccination of the general public.

**1. mRNA** (e.g., Pfizer-BioNTech, Moderna, CureVac AG, Tubingen, Germany, CanSino, Tianjing, China): Delivery of mRNAs to cells for making disease-fighting proteins has been under study for two decades [29]. This is the newest vaccine platform where mRNAs are encased in lipid nanoparticles for uptake by host cells. Once inside, host machinery is used to synthesize the spike protein or part of it. The mRNAs are then degraded, leaving no trace to cause potential deleterious effects. The most significant advantages of mRNA vaccines lie in the simplicity and rapidity of their design, the ease of its changes and production. The final products also contain a limited number of ingredients. The major disadvantage of mRNAs is their instability, requiring deep-freezing temperatures as low as −80 °C for storage and the necessity of prompt use after thawing. Administration of 2-doses weeks apart is also costly and cumbersome. Phase III clinical trials yielded 95% efficacy for the mRNA vaccines made by two different companies, providing a high level of confidence for such a new vaccine format.

**2. Adenovirus-delivery** (e.g., AstraZeneca (AZ) or Covishield; Johnson & Johnson (J&J) New Brunswick, NJ, USA; Sputnik-5, Moscow, Russia; CanSino, Tianjin, China): Adenoviruses have been used for many decades as a vehicle to deliver DNAs initially for gene therapy and then vaccines against a number of viral diseases [30]. How to render these viruses non-replicative and thus safe, how to package vaccine-coding genes into its genome, how to mass-produce them in mammalian cells (e.g., Vero, CHO, BHK-21), and how to purify the viruses have all been worked out in great details. These are the major advantages for adenovirus-delivery of vaccines. The urgency of the COVID-19 pandemic offers the unprecedented rapidity of completing Phase III clinical trials for these vaccines, yielding variable levels of efficacies up to 100% in preventing hospitalization, severe diseases, and death. The adenoviruses used are mostly those causing the common human cold, except for the AZ vaccines using a chimpanzee adenovirus (ChAdOx1). The requirement of a regular low temperature of ~4 C for storage of these vaccines is the distinct advantage over the mRNA vaccines. Single-dose administration of the J&J (ad26) and CanSino (ad5) vaccines, based on the outcome of Phase I&II and III trials for efficacy, offers additional advantages of cost-effectiveness and convenience for use in difficult to reach locations. Spunik-5 uses adenoviruses of two different serotypes (Ad26 and Ad5) by Gamaleya researchers for constructing the 1st and 2nd vaccine doses that are designed to avoid the secondary antibody response heightened against the 1st construct, as often seen in prime-boost vaccination scheme [31]. The major disadvantages of using adenoviruses for vaccine delivery remain to be concerns of their entry into the host cell nucleus as DNA viruses, raising the likelihood of their genomic integration and reshuffling, and reversion to pathogenicity.

**3. Recombinant protein-Nanoparticles** (e.g., Novavax, Gaithersburg, MD, USA): This is based on the commonly used strategy to produce vaccines as recombinant proteins in *E. coli* followed by their purification for display on novel nanoparticles for vaccination with adjuvants, e.g., Saponin-based Matrix-M1. The advantages of such vaccines include the use of well-developed biotechnologies and the precedent of FDA approval for similar vaccines as safe and effective for extensive use against other diseases. The disadvantage is the limited ability of peptide subunit vaccines to induce cytotoxic CD8 T-lymphocyte (CTL) responses. Phase III clinical trials of Novavax spike vaccines appeared to show ~90% efficacy overall and 100% against severe COVID-19.

**4. DNA** (e.g., Inovio, San Diego, CA, USA**):** This platform places mammalian expressible genes in DNA plasmids for transformation of and expansion in *E coli*. Isolated plasmids are delivered into cells of skin or muscles by using a device, which produces electric pulses to create membrane pores to let in the plasmids. Major advantages of DNA vaccines are the rapidity and simplicity of the vaccine designs, the ease of their changes, and mass production based on well-developed recombinant biotechnologies. Stability, easy storage, and cost-effectiveness are also the advantages of DNA vaccines versus mRNAs. The use of DNAs and the requirement of their nuclear entry raise the same concerns similar to those related to the use of adenoviruses or other DNA viruses. An additional concern is the consistency and efficacy of plasmid delivery via electric pulses. Preliminary reports of 2-dose clinical trials for spike DNA vaccines appeared to be safe and effective (>90%). Phase III trials of this DNA vaccine are still ongoing, but the outcome is expected to be ready for submission to FDA for EUA in 2022.

**5. Inactivated SARS-CoV-2 viruses** (e.g., Bharat Biotech, Hyderabad, India; SinoVac, Beijing, China; SinoPharm, Beijing, China). Many FDA-approved vaccines in current use against infectious diseases consist of the causative agents killed by chemical and/or physical means, e.g., formalin, UV/gamma irradiation, β-propiolactone, and binary ethylenimine. One advantage of using such a time-honored and well-established methodology for making COVID-19 vaccines includes the technical familiarity, facility availability, and reliability of production. Using a complete virus as the vaccine is also advantageous by having a full repertoire of all available viral antigens to optimize immunogenicity [32]. The disadvantages of this approach include the requirement of expensive and cumbersome P3 safety facilities for obtaining the pathogenic viruses and antigen denaturation by the harsh inactivation conditions used by necessity to ascertain the safety of their application. Both China and India opted to develop such COVID-19 vaccines [33]. Fully inactivated or “dead” viruses are administered in two doses weeks apart. The efficacy was reported to be 78% for India’s Covaxin and to vary from 50–90% for the Chinese versions, depending on the sites of Phase III clinical trials.

All vaccines discussed above, except the last one, are designed to elicit a human immune response for producing antibodies against a single target, i.e., spike protein, thereby inhibiting its binding to ACE-2 receptor to block cell entry of the virus (see Section 3.1.2).

**6. Alternative and improved vaccines**: Below are several examples from a dazzling array of different ideas for vaccine designs. It is contemplated to produce a billion doses of a “people’s vaccine of spike protein” (Peter M. Hotez) by using simple and low-cost technology similar to platform 4, but using yeast cells [34], as has been done to make Hep B vaccines for the last 40 years in India. Available facilities exist to mass-produce flu vaccines in chicken eggs. This is exploitable for COVID-19 by incorporating Newcastle Disease Virus with a highly stabilized spike protein (NDV-HXP-S), which can be engineered by available technologies to withstand heat and chemical denaturation [35]. Structural biology offers infinite potential to design next generation of vaccines. One example is the “2P spike” made by introducing two proline substitutions into the spike protein to keep its structure stable in the prefusion state, thereby significantly increasing its antigenicity for producing more effective neutralizing antibodies [36]. A first HLA-I immunopeptidome analysis of virally infected human cell lines identified T cell epitopes in out of frame viral S, N, and non-structural proteins, providing new vaccine designs to target infected host cells against variants [37]. Oral and nasal spray vaccines represent additional formats under investigation. The latter was designed to elicit better immune response at the site of infection.

B. Human leishmaniasis: In contrast, no vaccine of the modern formats has been made available for general use for this disease despite persistent efforts for decades. Easy success was initially predicted because a spontaneous or chemotherapeutic cure of human leishmaniasis is followed by patients’ life-long immunity. A complete list of past preclinical efforts and clinical trials was compiled in detail in 2011 [38]. Interests in developing vaccines for leishmaniasis have persisted, as indicated by a steady stream of reviews written on this topic [39,40,41,42,43,44,45,46,47,48,49,50]. Readers are referred to these reviews for the history of past successes and failures, and details of the current attempts. It suffices to mention the earliest format of effective vaccination and representative examples of the subsequent efforts.

**1. Leishmanization (vaccination with live *Leishmania*):** Inoculating healthy individuals with lesion-derived live parasites in a hidden site is the crudest form of vaccination for prophylaxis of simple cutaneous leishmaniasis (CL). This is known as leishmanization [51] and has been practiced for millennia to protect individuals from the potentially facial disfiguring CL in the Middle East and Central Asia. The vaccinated individuals develop lasting immunity after self-healing. Leishmanization is thus effective and is still practiced today using avirulent cultured promastigotes as the vaccines in Iran (Ali Khamesipour, personal communication). In central Asia, a large clinical trial of vaccination was carried out by Russian workers with another avirulent strain grown in vitro, resulting in good protection overall. This vaccine was abandoned due to rare incidences of severe skin infection. There have been many preclinical trials in animal models using genetically attenuated or crippled *Leishmania* by knocking out essential genes, which are important for cell growth, replication, or virulence [52,53]. None has been developed to reach the stage of Phase III clinical trials.

**2. Vaccination with killed *Leishmania***: Significant attempts were made under WHO sponsorship to carry out clinical trials using cultured promastigotes, which were autoclaved to ensure their safety before use. Autoclaved promastigotes were used at 100–400 ug per dose with or without alum or BCG [54]; F. Modabber Personal communication]. This vaccine format proved safe, but was abandoned for lacking efficacy after extensive trials in the Middle East, North Africa, and South America. Prophylactic activities have been shown in pre-clinical trials in animal models with cultured promastigotes, which were inactivated in vitro by other means, e.g., anti-leishmanial drugs, gamma-irradiation, and heat-inactivation. No clinical trials have been undertaken with *Leishmania* killed by these means.

**3. Subunit vaccines:** The best studied subunit vaccine consists of three antigens, which were initially identified by screening *Leishmania* expression library with convalescent sera of patients who recovered from visceral leishmaniasis (VL). A trivalent peptide vaccine was subsequently produced in *E. coli* as recombinant products of fusion proteins (LEISH-F1+MPL-SE). This vaccine proved effective when used with appropriate adjuvants to immunize susceptible animals against the challenges with virulent parasites in preclinical trials. Clinical trials of this vaccine provided evidence of its safety in Phase I and II [39,41,55,56], but showed only limited efficacy in Phase III [46], hence no further development for general use. ChAd63-KH is another anti-*Leishmania* vaccine, consisting of two antigens, i.e., kinetoplast membrane protein-11 (KMP-11) and hydrophilic acylated surface protein B (HASPB) in a Simian adenovirus. Its vaccinability is indicated by their pre-clinical trials individually and preliminary clinical trials for immunotherapy of Postkala-azar Dermal Leishmaniasis (PKDL) in Sudan [57]. Other subfractional vaccines examined include soluble and/or insoluble fractions of cultured *Leishmania* or their secretory products; and other recombinant products of immunologically active *Leishmania* antigens. Immunoprophylatic protection of susceptible mice was demonstrated, for example, by immunization with purified LPG, recombinant *Leishmania* antigenic proteins, e.g., gp63 (zinc proteinase), and *Leishmania*-receptor for activated kinase (LACK). Few have been submitted to clinical trials in dogs, resulting in the commercialization of four approved for sale in the market as vaccines for canine leishmaniasis, e.g., *Leishmania* gp63 and A2, a mixture of ribosomal proteins and secretory products of cultured parasites in combination with different types of adjuvants. Detailed evaluations of available data showed that their applications have not significantly reduced the transmission of canine and human leishmaniasis [58,59].

**4. Other vaccines:** Attempts are being focused on designing *Leishmania* DNA vaccines and vaccine cocktails of additional recombinant peptides with adjuvant for prophylactic and therapeutic measures [39,60]. The potential of sand fly saliva antigens for vaccination has been discussed [40,61].

The preceding passages provide the sharp contrast of COVID-19 versus leishmaniasis vaccines. Most advanced vaccines in multiple formats never deployed before were rapidly developed, produced, and approved for massive rollout to immunize a very large population against COVID-19 within one year. In contrast, no new vaccine has been made available for human leishmaniasis after sporadic attempts for many decades. Several ineffective vaccines are available due to commercial incentives for canine leishmaniasis, mainly in southern Europe.

#### 3.5.2. Clinical Trials

COVID-19: Massive government funding and expedition of rapid regulatory reviews have made it possible to ramp up combined Phase I and II trials with several hundred volunteers and hundreds of thousands for Phase III without sacrificing the stringency of the protocols.

Leishmaniasis: Clinical trials have taken the usual lengths of many years to complete.

Clinical trials of all drugs are expensive, laborious, and time-consuming, requiring expertise and support for manufacturing, logistic and clinical management, and regulatory approvals. Vaccine trials for infectious diseases face additional constraints for the necessity of recruiting volunteers available at the right time in the right place. To have vaccines ready for trials takes time when the incidences of the targeted diseases may become too low, hampering the recruitment of volunteers. The pandemic COVID-19 with a high transmission coefficient has undoubtedly contributed to the ease of volunteer recruitment and rapid conclusions of Phase III trials. Recent work provides supplemental approaches with the potential to streamline future clinical trials, as described in Discussion Section 4.6.

#### 3.5.3. Human Challenge Trials

COVID-19: This is also known as the controlled human infection model (CHIM), whereby healthy human volunteers are recruited for inoculation with pathogens which cause curable infectious diseases, mainly for testing novel drugs or vaccines. There are stringent guidelines to ensure the availability of effective treatments ready for use to terminate the infection at the first sign of an untoward outcome. The ethics of this approach has been the subject of considerable debate. The severity of COVID-19 is considered to outweigh the ethical concern of CHIM, calling for action in an open letter of 14 October 2020 that was addressed to the UK Health authority and signed by a group of >100 scientists and other professionals, including 15 Nobel laureates (https://www.1daysooner.org/uk-open-letter, 14 October 2020). Two UK projects were initiated in response to the call:

The first COVID-19 challenge study was supported by >$45 million from the U.K. government and led by Imperial College London infectious-disease researchers (https://www.bbc.com/news/health-56097088, 17 February 2021). The project started in March 2021 with a handful of volunteers isolated inside London’s Royal Free Hospital. The target subjects are 18 to 30-year-olds free of COVID-19 symptoms and from other risk factors such as heart disease or diabetes. The study aims include tests of how effective vaccines are in warding off infection and symptoms, and how participants’ immune systems respond to the vaccination. It is planned to expand the study to other sites nationwide.

Another study was started by Dr. Helen McShane, professor of vaccinology, Department of Paediatrics, Oxford University (https://www.ox.ac.uk/news/2021-04-19-human-challenge-trial-launches-study-immune-response-covid-19, 19 April 2021). Subjects were also recruited from those under 30 who have recovered from infection in previous clinical trials. Vaccines of various doses were administered for determining optimal immune response; probing the boundaries of human immunity and the effects of the virus on the body from the moment of reinfection. The outcome is expected to elucidate protection from previous illness to help fast-track new treatments and vaccines.

Leishmaniasis: CHIM has not been established for leishmaniasis, although there was interest to initiate this [62].

The significance of this effort is its initiation to define and demonstrate safety, paving the way for future investigation of more substantive issues. The emergence of SARS-CoV-2 variants is expected to complicate the interpretations of the results from these projects.

#### 3.5.4. Vaccine Rollout

COVID-19: Vaccine rollout is subjected to considerable constraints by the inherent inequality between the wealthy and the poor, as expected. While WHO’s COVAX program is designed to ease this inequality among different countries, its effectiveness is at the mercy of support from wealthy countries. AstraZeneca is the only private company committed to helping resource-poor countries by providing its vaccine at cost. Rapid immunization of the world population is recognized as a matter of necessity to bring the pandemic under control. This has been marred by the inequality of vaccine distribution due primarily to the excessive greed of vaccine companies for profits (except AstraZeneca) as well as to national and diplomatic interests, and racial discrimination of the wealthy countries known as “vaccine nationalism”, “vaccine diplomacy” and “vaccine apartheid”.

Leishmaniasis: There is no vaccine yet to roll out, precluding the emergence of confounding issues.

The inefficient rollout of vaccines negatively impacts their effectiveness by allowing pathogens like SARS-CoV-2 to replicate in the infected population, especially those unvaccinated for accumulation of potentially transmission-enhancing and/or immunity-resistant mutations. Indeed, there has been the emergence of such variants in successive waves from Alpha to Delta to Omicron and now Omicron subvariants.

#### 3.5.5. Socio-Economic Impacts on Vaccination Rate and Herd Immunity

COVID-19: The vaccination rate is susceptible, in addition to rollout inefficiency, to a range of known economic, social, and other factors, which have plagued all previous vaccination programs. There are “vaccine skippers” uninformed of vaccine availability or living in hard-to-reach locations; “vaccine hesitants” fearing needles, vaccine inefficacy or side-effects; “vaccine skeptics” doubting the necessity of vaccination; and “anti-vaxxers” fooled by the misinformation of vaccine-caused harms. The negative impacts of these groups have slowed the rate of full vaccination in the USA, leveling at ~60% for the vaccination-eligible population of 12 years old and older (since July 2021). Subsequent resurgence of viral transmission with the emergence of delta-variant speaks for the necessity of booster shots. FDA indeed issued EUA for boosters of approved vaccines initially for the vulnerable groups and then for all (November 2021), and for vaccination of children of the 5–12-year-old age group since October 2021. The overall vaccination rate had not increased substantially for the subsequent 6 months, i.e., 63.7% as of 24 January 2022. Effective mathematical modeling for the threshold of herd immunity has been hampered by these and by other negative factors, e.g., premature abandonment of transmission-averting measures (social distancing, mask-wearing, lockdown) and inefficiency of vaccination to block viral transmission of emerging variants with waning immunity of vaccinated and convalescent populations [63]. The best scenario predicted for the outcome of the vaccination programs is that COVID-19 persists as seasonal flu requiring regular boosters. This prediction remains uncertain, pending further evaluation of the pandemic trajectory data for the emerging variants.

Visceral leishmaniasis: Vaccination rates to reach herd immunity have been calculated in planning for vaccine development [46].

#### 3.5.6. Human Immune Response to Vaccination

COVID-19: Vaccine rollout for extensive use makes it possible to closely follow the timeline for the evolution of human immune response to the vaccination. Cohorts of vaccinated and unvaccinated groups, and pre-and post-vaccinated individuals, are available for comparative studies. There were initial reports of positive antigen- or spike protein-specific B cell or antibody and T cell responses [64,65,66,67,68,69]. The sera from vaccinated and convalescent sera were both found to have antibodies with variable titers of viral neutralization activities in vitro, as noted for anti-spike monoclonals used for immunotherapy. Variable activities were further noted with reduction against certain highly contagious variants (e.g., E484K mutation), explaining post-vaccination breakthrough cases [70,71].

Further investigation of subjects ~6 months post-vaccination revealed waning IgM and IgG specific antibodies, although memory T cells and memory B cells were detected [72,73,74]. These interim studies nevertheless project an optimistic outlook for the mRNA spike vaccines having long-lasting potential. Human immune responses to infection and vaccination are inherently difficult to calibrate due to their shifting dynamics and individual variabilities. The complexity of these events is further complicated by the successive emergence of antigenically different new variants.

Leishmaniasis: The absence of vaccine rollout provides no similar studies to assess post-vaccination human immune responses. T cell immune response has been shown in patients long after cure, accounting for their lasting immunity against re-infection.

#### 3.5.7. Rare but Serious Side-Effect of Live Adenovirus Vaccines?

Vaccine-induced immune thrombotic thrombocytopenia (VITT)—A rare blood clotting disorder emerged on the order of 10^−5^ for AZ vaccines in Europe and 10^−6^ for J&J vaccines (8 cases in 7 × 10^6^), resulting in several deaths in the USA. The symptoms were manifested 2 weeks after vaccination, starting with severe persistent headache, leg swelling, persistent abdominal pains, blurred vision, tiny blood spots under the skin. Blood clots block veins draining blood from the brain. Sufferers are under 30 healthy young women (18–49 years old), producing antibodies that activate or attack their own platelets, resulting in their depletion [75]. Very few patients died of hemorrhagic brain stroke due to failure to restore platelets. This incidence caused weeks of “vaccine pause”, aggravating “vaccine skepticism”. The common denominator for both AZ and J&J vaccines is the use of adenovirus to carry the spike gene, raising the suspicion of this virus as the trigger (according to Dr. Eleanor Riley, Immunology/Inf Dis, Univ Edinburgh (https://www.reuters.com/article/instant-article/idCNL8N2M64C5, 13 April 2021). However, a temporary lowering of platelets was reported previously for Measles, Mumps, and Rubella (MMR) vaccination and therapeutic use of mRNAs. According to European Medicines Agency (EMA)/EU Health & Food Safety Commission, AZ vaccines have been used in 27 EU countries for a population of 448 million. The use of AZ and J&J vaccines was limited to the older age group (>55) at one point to avoid such a rare incidence of VITT.

### 3.6. Leishmania Platform for Safe and Effective Delivery of Vaccines

#### 3.6.1. *Leishmania* as an Effective Vaccine Platform

This is suggested by the fact that most patients of leishmaniasis develop life-long immunity after a spontaneous or chemotherapeutic cure. Indeed, memory T cells were reported to persist in subjects long after a full recovery from cutaneous and visceral leishmaniasis, as they are functionally activatable in vitro with *Leishmania*-specific antigens [76,77]. Clinically, the best example is the simple cutaneous leishmaniasis (CL) endemic to central Asia and the Middle East. There, Leishmanization with live *Leishmania* (see Section 3.5.1, II-1) has been practiced as the crudest form of vaccination, reminiscent of “variolation” to prevent smallpox. Anecdotal histories of both practices suggest a common root of origin from ancient countries in the East. While leishmanization has been continuously practiced in some locations until today, variolation was abandoned long ago. Both are relatively effective, but the margin of their safety differs significantly. Variolation has a death rate of 2–3% plus incomplete protection from re-infection [78]. In contrast, leishmanization has survived the test of time not only for efficacy but also safety by causing no more than simple CL—a non-lethal, often insignificant and self-healing skin infection.

Notably, the development of life-long immunity is the exception rather than the rule after recovery from most infectious diseases. Besides leishmaniasis, another example is Q fever in animals and humans caused by a Gram-negative bacterium*, Coxiella burnettii* [79,80]. Recovery from a bout of Q fever renders patients forever immune to this disease, as seen in leishmaniasis. The causative agents of these two different diseases are phylogenetically distant but share considerable similarities in the biology of their parasitism. Both *Leishmania* and *C. burnettii* utilize macrophages as their exclusive host cells by residing in their acidic phagosome-lysosome vacuolar system. Patients who recovered from both diseases are also marked by a strong and lasting antigen-specific DTH (delayed-type hypersensitivity), indicative of the development of T cell-mediated immunity. Natural infection by these etiological agents thus amounts to successful vaccination, indicative of their possession of not only effective vaccines but also functional adjuvants in the context of vaccinology. Of relevance is adjuvanticity, as defined in its broadest sense. Both organisms are endowed with resistant surface glycoconjugates. While different in architecture, they function similarly to protect themselves and, by inference, the natural vaccine molecules they contain from losses, for example, to degradation by lytic factors present in the animal body fluids. The parasitism of macrophages by these agents further brings their endogenous vaccines to the desired destination of the antigen-presenting cells (APC). The subsequent entry of both microbes into the phagosome-lysosome vacuoles also favorably places their vaccine molecules in position to facilitate their proteosomal and lysosomal processing for presentations via MHC Class I and Class II pathways for T cell activation. In contrast to *C. burnettii* as a select agent highly virulent with a resistant phase, *Leishmania* is a biosafety level II pathogen and thus a safer choice for development as a vaccine platform. Additional advantages of *Leishmania* include in vitro cultivability of these protozoa in a chemically defined medium free of animal sera for mass production in fermenters and its well-developed transgenic technology to express foreign vaccines with ease [81].

#### 3.6.2. *Leishmania* are Made Safe as a Vaccine Platform by Installation of a Dual Suicidal Mechanism for Singlet Oxygen-Initiated Inactivation

Live *Leishmania* can be an effective vaccine platform, as shown by their use for Leishmanization (see Section 3.6.1). As such, its clinical applications have limits. For example, leishmanization results in the formation of skin lesions, which may on rare occasions become protracted, requiring weeks to heal even in healthy individuals with apparently intact immune systems. Leishmanization is off-limits to those who are immunologically deficient congenitally or due to immunosuppressive infections, e.g., HIV or under immunosuppression after organ transplantation.

**Figure 1:**
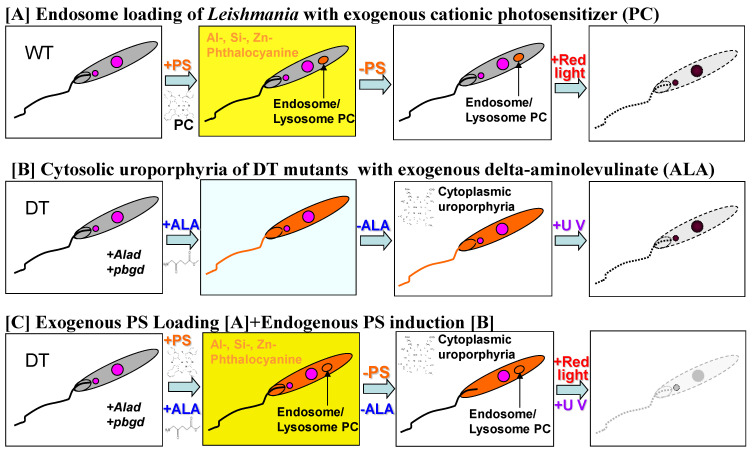
Schematic depiction of endosomal and cytosolic loading of *Leishmania* with different photosensitizers for complete inactivation by light exposure. (**A**) Photosensitizers (PS) used include phthalocyanines (PC) with different coordinating metals (Al, Si, Zn), which are engineered during chemical synthesis to have different axial ligands, such as amino groups for cationicity. Wildtype (WT) *Leishmania* take up these cationic PCs (+PS) in vitro by endocytosis into their endosome/lysosome vacuoles, rendering them sensitive to red light inactivation (see Section 3.6.2). (**B**) *Leishmania* doubly transfected with mammalian cDNAs (DT) to express the 2nd and 3rd enzymes (+Alad,+pbgd) in the heme biosynthetic pathway undergo uroporphyrinogenesis, i.e., production and accumulation of xenogenic uroporphyrin 1 in the cytosol when exposed to the products of the 1st enzyme in this pathway, i.e., delta-aminolevulinate (+ALA) (see Section 3.6.2). Exposure of the uroporphyric *Leishmania* to longwave UV results in their rapid inactivation. (**C**) Double loading of *Leishmania* with a combination of cationic amino-phthalocyanine exogenously [A] and uroporphyrin 1 endogenously [B] ensures their photosensitization for complete inactivation on light exposure.

A new approach was devised to render *Leishmania* totally non-viable and thus non-disease-causing with minimal loss of their adjuvanticity. Their use as a vaccine platform is thus not only effective but also made safe. A dual light-activatable suicidal mechanism was installed in *Leishmania* to achieve this (Figure 1A–C). We first exploited its natural genetic deficiencies of missing the genes coding for the first five enzymes in the pathway for porphyrin/heme biosynthesis. Promastigotes were genetically engineered in vitro with mammalian cDNAs to express delta-aminolevulinate dehydratase (ALAD) and porphobilinogen deaminase (PBGD), the 2nd and 3rd enzymes in the heme biosynthetic pathway [82,83,84]. These transgenics accumulate UV-sensitive uroporphyrin I cytosolically when exposed to delta-aminolevulinate (ALA)—the product of the 1st enzyme in this pathway. The absence of downstream enzymes after PBGD accounts for the cytosolic accumulation of uroporphyrin I to a very high level. The uroporphyric *Leishmania* were then further loaded endosomally with a red-light sensitive phthalocyanine, which was chemically engineered for cationicity to facilitate endocytic uptake [85,86]. Light excitation of these *Leishmania* cells loaded with both dyes results in generating abundant singlet oxygen (^1^O_2_) in both cytosolic and vacuolar compartments [82,87]. These radicals are highly reactive to oxidatively inactivate cellular proteins, lipids, and nucleic acids. However, only those in the immediate vicinity of ^1^O_2_ generation are affected due to the extremely short lifespan of these radicals (half-life in microseconds). Indeed, the ^1^O_2_ generated endogenously in the cells vanishes too rapidly to travel across the plasma membrane, thereby leaving the surface glycoconjugates intact to serve the adjuvant functions, as described (Section 3.6.1). Similarly, *Leishmania* are not susceptible to extracellular ^1^O_2_ generated in the milieu, accounting for the necessity of their dye-loading followed by light excitation to produce intracellular ^1^O_2_ for effective inactivation. The reactions of ^1^O_2_ with intracellular biomolecules trigger the generation of other reactive oxygen species (ROS) secondarily, including hydroxyl radicals (OH^.^), peroxides (H_2_O_2_), and superoxides (O_3_). The contribution of these ROS to the inactivation of *Leishmania* is considered limited on account of their neutralization by a multitude of highly active endogenous antioxidant enzymes. These antioxidants are known to exist abundantly in *Leishmania*, e.g., peroxidases, superoxide dismutase, peroxiredoxin, and trypanothione reductase. *Leishmania* neither produce ^1^O_2_ nor encounter these radicals in the environments of their natural life cycle. Hence, they have not been subjected to selective pressures to evolve a specific mechanism for ^1^O_2_ detoxification. Only plants are known to have evolved the potent mechanism by strategic placement of beta-carotene and tocopherol to scavenge ^1^O_2_ produced in the chloroplasts during photosynthesis. The absence of this or other potent free radical scavenging mechanisms in *Leishmania* accounts for their sensitivity to internally generated ^1^O_2_ as the principal inactivating agent. The manifestation of this inactivation is vividly illustrated by the sudden cessation of flagellar motility when dye-loaded *Leishmania* were exposed to a dim light of illumination during observation by phase-contrast microscopy [82,87]. The dual suicidal mechanism installed in *Leishmania* affects their complete inactivation, as shown by their inability to grow in culture medium, to survive in macrophages in vitro, and to produce lesions in susceptible animals [88].

#### 3.6.3. Safe and Effective Delivery of Vaccines by ^1^O_2_-Inactivated *Leishmania* for T-Cell Mediated Immunity Against Infectious and Malignant Diseases

**Figure 2:**
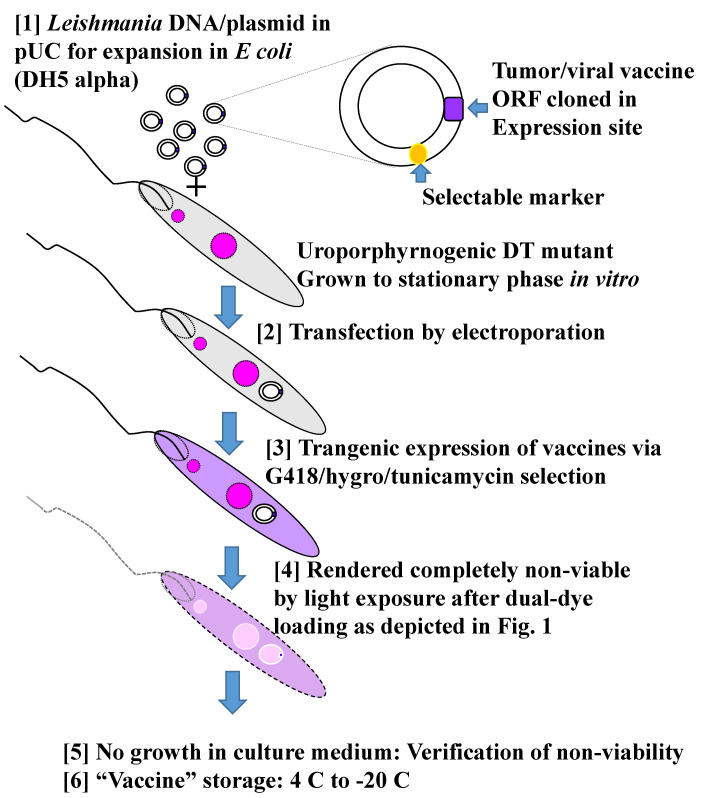
Schematic depiction of transgenic production of vaccines in DT uroporphrinogenic *Leishmania* and their complete inactivation by dual dye-loading followed by light exposure. See Figure 1 for ALA-induced cytosolic accumulation of uroporphyrin 1 in DT *Leishmania* and loading of their endosomes with cationic phthalocyanine. Workflow: [**1**] Clone cDNAs of tumor/viral vaccines into the expression site of a *Leishmania/E. coli* shuttle vector for expansion in DH5-alpha and grow uroporphyrinogenic DT mutants of *Leishmania* under tunicamycin (TUN) and G418 selection; [**2**] Transfect the DT mutants with the plasmids isolated by electroporation; [**3**] Select transfectants with hygromycin (HYG) for vaccine expression; [**4**] Place the transfectants under selection with HYG+TUN+G418; [**5**] Expose the transfectants to amino-phthalocyanine (PC) for endosomal uptake and to ALA for cytosolic accumulation of uroporphyrin I followed by longwave UV + Red light exposure for ^1^O_2_-initiated inactivation (see Figure 1); [**5**,**6**] Verify complete loss of viability of the inactivated cells and their storage by means, as stated.

Vaccine preparation for this is depicted in Figure 2. The initial evidence for this was provided by exposure of dye-loaded *Leishmania* to light for ^1^O_2_-inactivation before and after their loading of antigen-presenting cells (APC), e.g., macrophages and dendritic cells (DC) in vitro [84,85,86]. The uroporphyrinogenic mutants load macrophages as readily as the wildtype control, indicative of their full efficacy in delivering vaccines to APC. Exposure of the mutant-laden macrophages to ALA induced a transient porphyria of these host cells when using cell lines, e.g., J774, but not primary mouse macrophages. In both cases, the intracellular mutants become highly uroporphyric and persistently remain so. A host-parasite porphyric disparity is thus created so that light exposure of these macrophages after infection selectively inactivates *Leishmania* for disintegration in the parasitophorous vacuoles (PV), leaving the host cells unscathed. The disappearance of the PV ensues in these cells, concomitant with their return to normalcy from *Leishmania*-mediated immunosuppression, as shown by microarray analysis of their global transcription profiles [89]. Specifically, sequestration of MHC molecules seen by live *Leishmania* to the PV is reversed, returning them to the normal diffused pattern [89]. This is accompanied by a switch in their cytokine profiles from immunosuppression to immunity [84]. Delivery of vaccines to APC is equally effective with *Leishmania*, which is pre-inactivated by dye-loading followed by light exposure before use for APC loading [84]. This approach ensures a complete inactivation of *Leishmania* assessable before use to guarantee their safety.

Immunization of susceptible animals with ^1^O_2_-inactivated *Leishmania* by either approach prophylactically protects them against visceral and cutaneous leishmaniasis. The endogenous natural vaccines and adjuvants thus remain active after this oxidative means of inactivation. Hamsters were immunized first with live uroporphyric *Leishmania,* followed by their inactivation in situ via illumination of the immunization sites with an external light source [90]. No live *Leishmania* is demonstrable at the site of injection. The animals immunized were protected from challenges with virulent *L. donovani*. Cytokine transcript analysis of splenic cells provided evidence consistent with the expected protective phenotypes of Th2- to-Th1 conversion. The immunity acquired by the photodynamic vaccination is adoptively transferable to naïve hamsters with splenic cells from immunized animals. In a separate study, immunization of BALB/c mice with pre-inactivated *L. amazonensis* via the duel suicidal mechanism described protected them against homologous challenges [91]. The lesion onset was significantly delayed, and the parasite loads were substantially smaller. This level of protection is highly significant, considering that BALB/c mice are highly susceptible to experimental leishmaniasis.

The potential use of ^1^O_2_ pre-inactivated *Leishmania* as a carrier to deliver foreign vaccines was initially shown by using *Leishmania* genetically engineered to express ovalbumin (OVA) as the T cell antigen [85]. Antigen-presenting cells loaded with ^1^O_2_-inactivated OVA-expressing *Leishmania* process them effectively to co-present the OVA CD8+ T cell-specific epitope (SIINFEKL) with MHC Class I molecules. The APCs so primed were shown to activate a T cell line specific to the homologous peptide epitope. This positive outcome with the effective delivery of a model T-cell antigen by the inactivated *Leishmania* prompted us to examine candidate vaccines for malignant and viral diseases. Candidate vaccines chosen were expressed by episomal transfection of porphyrinogenic *Leishmania* with their cDNAs. The expression of these cDNAs in the transfectants was shown at transcriptional levels by RT-PCR, e.g., EBOLA GP [92] and, in most cases, at protein levels by Western blot analysis, e.g., HIV GP120, SARS-CoV-2 spike and membrane proteins, melanocytic PRAME, CAGE cancer antigens, and enolase 1 (ENO1) of lung and pancreatic cancers. All transfectants obtained remain stable and susceptible to ^1^O_2_ inactivation to completion by the duel suicidal mechanism, as descrived (see Section 3.6.2).

Immunization of experimental mouse models with the vaccines delivered by ^1^O_2_ inactivated *Leishmania* produced encouraging preliminary results in two separate studies. One is the immunotherapeutic potential of the inactivated ENO1 transfectants [93]. Vaccination of BALB/c mice with these inactivated transfectants after implantation of murine ENO1-positive lung cancer cells prevented the development of otherwise massive tumors in their peritoneal cavity. This immunity is adoptively transferable with splenic cells to significantly reduce the tumor development of human ENO1 lung cancer cells in immunosuppressed mice. A similar but less dramatic effect of such vaccination was noted against tumor development in the murine pancreatic cancer model.

Another example is the preliminary observations of the investigation still in progress with inactivated *Leishmania* expressing SARS-CoV-2 spike and membrane proteins. In two independent studies, immunization of C57BL/6 mice was shown to elicit the generation of CD4+ and CD8+ T cells responsive to activation by the peptide antigens in vitro. These immune cells are specific to the viral antigens, as clearly indicated by the completely negative readings of the control transfectants without the transgenes. This is remarkable, considering that both spike and membrane transgene products must be rather small in quantity versus the large amounts of many different *Leishmania* proteins, i.e., up to 8000 proteins known or expected from the total ORFs of the *Leishmania* genome (see Section 3.1.1). These viral antigen-specific T cells are expected to have functional significance, pending investigation in a suitable mouse model for COVID-19.

## 4. Discussion

### 4.1. Prospect of Disease Control from the Perspective of COVID-19 Pandemic by Vaccination and Beyond

What transpired most clearly from this comparative analysis is the possibility of making multiple vaccines available for use very quickly in an emergency, like the COVID-19 pandemic. Precedence is thus set for the potential to do the same, in principle, for all infectious diseases, preferably before they turn epidemic or even pandemic. There is evidence that global warming accelerates this event for many diseases [94], including leishmaniasis with the sign of its northward expansion. Leishmaniasis is just one of the 20 different diseases targeted by WHO for elimination. While none is pandemic, together they cause significant mortality and morbidity. The incidences of these diseases are limited mainly to low- and-middle income countries (see Section 3.3.3), making it unlikely to garner the kind of lavish support as Operation Warp Speed (see Section 3.4.4). The necessity of developing vaccines against all these diseases is, however, evident [95], especially for zoonoses, like leishmaniasis or those emerging periodically from wild animals, like COVID-19 (see Section 3.3.1). All infectious diseases with wild animal reservoirs are impossible to eradicate, requiring constant attention to mitigate their resurgence by costly preventive measures (see Section 3.4.3) and surveillance (see Section 3.4.1). Vaccine development for these diseases becomes all the more urgent due to the lack of effective drugs and drug resistance (see Section 3.4.2 A–C).

While all events associated with anti-Covid vaccines are still unfolding, indicative of the enormity and uncertainty of such undertaking, invaluable information has been made available at a rapid pace to inform those who are interested in such a bench-side to bed-side endeavor for fighting diseases. Also made evident by the pandemic-related work is the relatedness of vaccination to many facets of the disease, requiring expert input from disparate disciplines in line with the concept of the “One medicine, One health, One-world” approach. Attempts are thus made by covering all pertinent aspects of both diseases for comparison with the recognition of inevitable omissions. In the same vein, topics for further discussion are broad-based but selective to highlight and elaborate specific points of interest.

### 4.2. The Success of Anti-Covid Vaccines Provides Impetus to the Development of New Approaches in Vaccinology 

The new approaches include *Leishmania* Vaccine Platform (see Section 3.6.1, Section 3.6.2 and Section 3.6.3). The mRNA and adenovirus for delivering vaccines (see Section 3.5.1 A) are both a novelty, although they are based on biotechnologies after refinements by extensive investigation for decades [29,30]. Mono-specific vaccines of both platforms were produced, solely containing the spike protein of SARS-CoV-2—an apparently ideal vaccine target, considering the critical role it plays in the entry of virus into the host cells (see Section 3.1.2). Indeed, vaccinated individuals were shown to produce infection-blocking anti-spike antibodies, which were found, however, to decrease in titers with time and in activities against emerging mutants (Section 3.5.6) (see Section 4.4 for further discussion). Specifically, both Delta and Omicron variants were found to replicate in and disseminate for transmission from the vaccinated. However, they were still protected from severe illness, hospitalization, and death, suggestive of the development of antibody-independent immunity. The absence of pulmonary pathology among the vaccinated is thus thought to result from the activities of vaccine-activated CD8+ T cells, which swoop in to lyse virally infected cells, thereby limiting the infection to the upper respiratory tracts. If so, *Leishmania*-based delivery of spike protein has the potential to strengthen such CTL activities for lasting immunity, as discussed (see Section 3.6.3).

### 4.3. The Successful Deployment of Adenoviruses as a Platform for Anti-Covid Vaccines Makes the Use of Leishmania for Vaccine Delivery all the More Conceivable

Various adenoviruses genetically engineered to cripple their replication have long been explored as vaccine carriers against a myriad of significant diseases, e.g., SARS, Ebola, Zika, influenza, AIDS, tuberculosis, and malaria [96]. The COVID-19 pandemic offered the best opportunities for clinical trials to assess various adenovirus carriers, e.g., J&J, AZ, Sputnik-5 (see Section 3.5.1 A2), not only for their safety but also their efficacy. *Leishmania* rendered non-viable by installing a double suicidal mechanism (see Section 3.6.2) compares favorably in safety margin versus the live albeit replication-deficient adenoviruses. While *Leishmania* are far more complex than viruses structurally and composition-wise as eukaryotic protozoa, the mode of their parasitism is highly specific and marked by many elements of subtleties and stealth. Indeed, leishmaniasis is thought to have evolved from a long history of host-parasite interactions for their mutual adaptations. This is suggested by the extant niche of *Leishmania* endoparasitism, alternating between the sand fly guts and the phagosome-lysosome vacuoles of mammalian macrophages (see Section 3.1.2 and Section 3.3.1). *Leishmania* exploit the endocytic mechanisms of these phagocytes for entry as their exclusive host cells, taking reclusive residence in their parasitophorous vacuoles; in contrast, pathogenic viruses, like SARS-CoV-2, break into multiple cell types and take over their biosynthetic machineries for viral replication (see Section 3.1.2). The adenoviral vectors are rendered non-replicative but still deliver vaccines in a similarly invasive way. *Leishmania* are non-toxigenic pharmacologically and elicit no acute host immune response, and so, much as certain cutaneous species, have been used as live cells for human vaccination in Leishmanization (see Section 3.5.1 B). Such immunogenic and adjuvant properties of *Leishmania* can now be preserved by genetic and chemical engineering to render them totally non-viable by light-inducible ^1^O_2_ inactivation (see Section 3.6.2). These leishmanial vectors thus possess favorable features of safety and efficacy for vaccine delivery.

### 4.4. The ^1^O_2_-Inactivated Leishmania are Potentially Deployable as a Platform to Deliver Multiple Vaccines from a Single or Multiple Pathogens Simultaneously Against Different Diseases

*Leishmania* have a large genome to accommodate foreign genes encoding desirable vaccines episomally or chromosomally and possess the eukaryotic biosynthetic machinery for their efficient transcription, translation, and post-translational modifications (see Section 3.1.1). *Leishmania* thus can express a large set of immunologically active peptide epitopes of a giving pathogen, e.g., those identified for SARS-CoV-2 [97,98]. Coupled with the natural vaccines known to exist endogenously in *Leishmania*, such transgenic is a de facto chimeric vaccine suitable for immunization against both COVID-19 and leishmaniasis. The ^1^O_2_-inactivation of such a chimeric vaccine is equivalent to, but better than, combining both pathogens inactivated separately by chemical or physical treatments (see Section 3.5.1 A5). Inactivation of whole pathogens by these means invariably diminishes their vaccinability, as rigorous conditions are often required by necessity to ascertain safety.

### 4.5. Complementary to the Current Mono-Specific Anti-Covid Vaccines Is Perhaps the Use of Leishmania Transfectants Expressing Spike Protein Together with One or More Additional SARS-CoV-2 Antigens

Waning immunity and breakthrough cases of those immunized with the available monospecific vaccines are accountable, for the most part, by the mutations of the spike proteins in the emerging variants. This is well-known concerning molecules of all pathogens at the interface of host-parasite interactions. Frequent mutations of the spike protein increase the chance of their selection by immune pressures for the best fits to evade humoral immunity. Indeed, SARS-CoV-2 variants (Section 3.1.3) have emerged successively with an increasing number of mutations over the earlier versions in their spike proteins, e.g., up to 18 in the Delta-variant and ≥32 in the Omicron-variant. The Delta-variant became less susceptible to neutralization by previously made spike protein-specific monoclonal antibodies and by polyclonal antibodies in the sera from mRNA vaccinated cohort [99,100], accounting likely for the ease of its transmissibility or contagiousness. The Delta-variant infection was indeed reported to produce elevated viral loads by as much as 10^3^ fold higher than those produced by the prior variants. The Omicron-variant is predicted to be even more transmissible and more vaccine-resistant, as reported from the preliminary studies by the British government scientists.

Mutations of spike proteins alone do not fully explain all the events observed, e.g., no severe clinical sequelae despite the heavy viral loads and the emergence of inflammatory immunopathology long after the infection is over (Section 3.2.1). These phenomena are reminiscent of the *Leishmania* model for virulence [101,102], depicting separate groups of molecular determinants independently responsible for distinct phases of disease progression from infection to immunopathology to resolution. Independent up- and down-regulation of these functionally different molecules may thus produce a spectrum of clinical manifestations from asymptomatic to mild to severe cases in discordance with the pathogen loads [101].

Vaccination with *Leishmania* delivery of multiple antigens has the potential to foster the effectiveness in preventing the development of all signs and symptoms of COVID-19, irrespective of spike protein mutations. Of particular interest is to determine if a single dose of a multivalent vaccine is sufficient to elicit lasting immunity by this approach, mimicking the outcome of leishmanization (see Section 3.5.1 B). The emergence of mutants in successive waves seen with increasing resistance levels appears to make the vaccine formulation with spike protein alone unsustainable. Simply administering more booster shots of the same or sequence-revised vaccines is not expected to outrace the force of natural selection for the best-fit spike protein. There is, in fact, no scientific rationale against the development of multivalent vaccines from the very beginning, regardless of the platforms used.

### 4.6. COVID-19 Pandemic Has Brought Several Important Areas of Vaccine-Related Research into Focus for Attention

(1) Foremost perhaps is the significance of the ongoing active investigation of clinical samples for elucidating human immune response to SARS-CoV-2 infection and vaccination (see Section 3.5.6). Such efforts provide crucial information for real-world verification of the laboratory findings obtained at the cellular level in vitro and with animal models in vivo.

(2) The initiation of “Human challenge trials” is made possible by the urgency of the COVID-19 pandemic (see Section 3.5.3), akin to controlled human infection model (CHIM) already used to screen drugs/vaccines for their safety and efficacy against some infectious diseases [103]. The pandemic urgency may further incentivize technical breakthroughs to further reduce the risk of this model for regulatory compliance so that it can be used to study areas of significance directly related to human infection and immunity. One area of special interest for such investigation in CHIM is the mechanism of animal-to-human transmission of pathogens. The outcome is expected to shed light on how different pathogens jump from animals to humans relevant to the source of their origin, e.g., SARS-CoV-2. Such investigation also has the potential to explain different outcomes of pathogens’ animal-to-human transmission, e.g., highly airborne transmissibility of the virus to cause catastrophic pandemic COVID-19 versus *Leishmania*’s vector-transmission to cause wide-spread endemics of cutaneous leishmaniasis with limited severity (see Section 3.3.1). Another area of interest to study in CHIM (and in clinical trials) is the post-chemotherapy-immune response of the volunteers to the therapeutically killed pathogens in situ. Since no drug or treatment is expected to reach everywhere in the body to eliminate all targeted pathogens, immune clearance of the residual pathogens has long been considered a mandatory step for the “clinical cure” of infectious diseases after therapeutic intervention. The outcome has the potential to help design vaccines and drugs that are more effective than those in use (see Section 3.4.2 A). Pathogens assessed as weak in vaccinability may have them strengthened by using *Leishmania*-based delivery of their vaccine candidates (see Section 3.6.1,Section 3.6.2 and Section 3.6.3).

(3) Of utmost importance are several unanswered questions relating human immunity to pathogenic infection, i.e., exactly how pathogens, like SARS-CoV-2 or *Leishmania*, initiate human infection to cause their respective diseases, how the vaccines designed against them elicit a human immune response, and why the longevity of immunity against re-infection varies. It has long been thought that the outcome of human immunity to infection is determined at the first moment of host-pathogen/vaccine interactions and the dynamic events after the first encounter at the cellular and molecular levels in situ. These events have defied scrutiny in the real world. Real-time imaging and time-lapsed sample collections present technical and ethical hurdles for investigation in CHIM.

The closest available model for such studies is the in vitro simulation of SARS-CoV-2 infection by lung organoid chip/microfluidic approach [25]. The novelty of this model is the design with an artificial basement membrane lining with epithelial cells outside facing the airway passage and with endothelial cells inside facing the microfluidic blood flow. The introduction of SARS-CoV-2 to the airway initiates the infection used for studying its progression and also the response to various soluble factors and immune cells loaded to the circulating fluid phase. In vitro differentiation of pluripotent stem cells provides different cell types needed for such models. Microfluidic organs-on-a-chip of 3D cell cultures are available for other tissues. They are suitable for investigating pathogens with different portals of entry, e.g., skin, liver, colon, and brain. Time-lapsed imaging is possible, and samples are collectible from such 3D models for omics studies. Meta-analysis of the omics data is within reach by computation with the assistance of AI and machine learning programs. Information from such organoid models can integrate with one another, CHIM, and patients’ clinical data to elucidate human infection and immunity.

It is feasible to apply this approach for elucidating individual variabilities to specific infections/vaccination. This is ultimately in the domain of research for personalized medicine. Acquisition of information from individualized 3D-organoid models is highly desirable. The outcome will have considerable potential to define individual differences in responses to a given infection or vaccine, thereby explaining the wide spectrum of disease clinical signs and symptoms (see Section 3.2.1 and Section 3.2.2) and uncovering unforeseen rare, but severe, consequences of vaccination, e.g., VITT (see Section 3.5.7). The accomplishment of such feats in the future is thus expected to delineate individual disposition of a given infection, i.e., asymptomatic, mild cases, severe illness or death, as well as to vaccination, e.g., intensity and longevity of the immunity. Inching toward that aim is perhaps recent progress in completing human genome sequencing whereby >100 additional protein-coding ORFs were identified [104]. Considerable more advances are still needed for contemplating the approach mentioned toward a full understanding of individual variations in infection and immunity.

Acquisition of knowledge in human infection and immunity is indispensable to provide precise information so that scientific experts, healthcare officials, and political leaders can make the right policies rapidly and accurately for vaccination of the population and to convince the general public of its value to combat vaccine hesitancy, skepticism, and anti-vaxxerism (see Section 3.5.5).

## 5. Conclusions

While COVID-19 is a disastrous pandemic, it has energized the scientific community to rise to the occasion by making tremendous progress and contributions to blunt its ferocity, if not yet stop it completely. All past pandemics ended after running their courses, presumably via the selection of the best-fit hosts with more robust immunity and of pathogens for attenuation toward avirulence. The scientific victory to aim for is thus not to stop the pandemic, but to mitigate or minimize its destruction, for example, the human death tolls. By that measure, remarkable progress has been made by lowering the pandemic death of the world population to 0.07% for Covid-2019 (5.62 million/~8 billion as of 26 January 2022) from up to 5.4% for the 1918 Spanish flu (17–500 million), and up to ~50% for the Black Death during the 14th-century Medieval period and the Byzantine Empire (541 C.E.). What has contributed most is undoubtedly the availability of multiple anti-Covid vaccines completed in record time for world immunization. The mRNA vaccines capture the brightest aura in the spotlight as expected in this era of synthetic biology. Still, all anti-Covid vaccines await the test of time for their true effectiveness, like all vaccines previously developed and deployed. The persistence of COVID-19 serves as a reminder for the imperfection of the vaccines in use (Section 3.5.1 A). Worthy of further exploration is thus all alternatives in the pipeline, including ^1^O_2_-inactivated *Leishmania*, as a novel platform for safe and effective vaccine delivery (Section 3.6.1, Section 3.6.2 and Section 3.6.3). They offer distinct advantages despite their manufacturing complexity. To offset this drawback, such products may be viewed in the context of “nature being a much better manufacturer” for “organic produce”, which is highly valued despite its higher cost to produce.

The voluminous literature generated as a result of this pandemic has stimulated the spirits of all, including leishmaniacs, by providing multi-directional roadmaps to follow. Some areas of interest for the future were discussed from our perspectives in vaccinology.

## Data Availability

Not applicable in the absence of original data.
